# Supplementary material for: Chitosan-Strontium Oxide Nanocomposite: Preparation, Characterization, and Catalytic Potency in Thiadiazoles Synthesis
Source: Polymers (Basel). 2022 Jul 12;14(14):2827. doi: 10.3390/polym14142827 (PMC9322490; doi:10.3390/polym14142827)
Supplement: Supplementary file 1 [file polymers-14-02827-s001.zip › polymers-1807842-supplementary.pdf]

Supporting Data

Chitosan-strontium oxide nanocomposite: Preparation, characterization, and catalytic potency in thiadiazoles synthesis

Khaled D. Khalil, Sayed M. Riyadh, Nazeeha S. Alkayal, Ali H. Bashal, Khadijah H. Alharbi, Walaa Alharbi

Figure S1. Analytical data of Compound 5a

^{1}H NMR of Compound **5a**

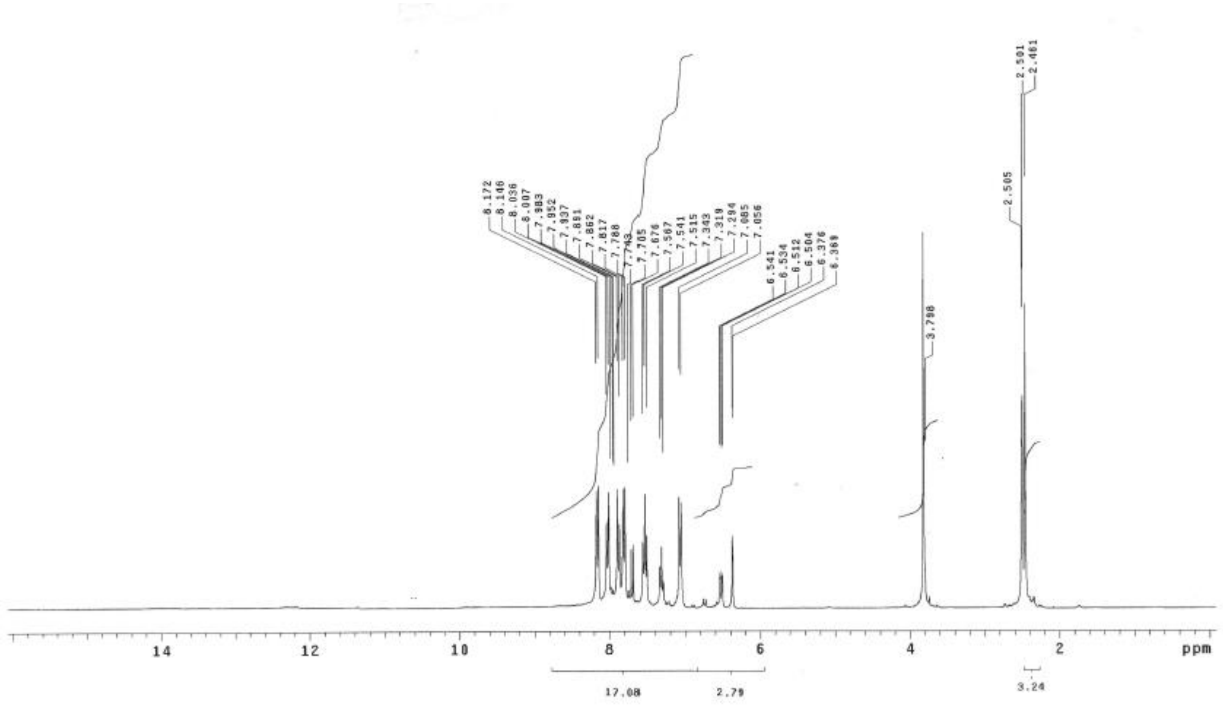

^{13}C NMR of compound **5a**

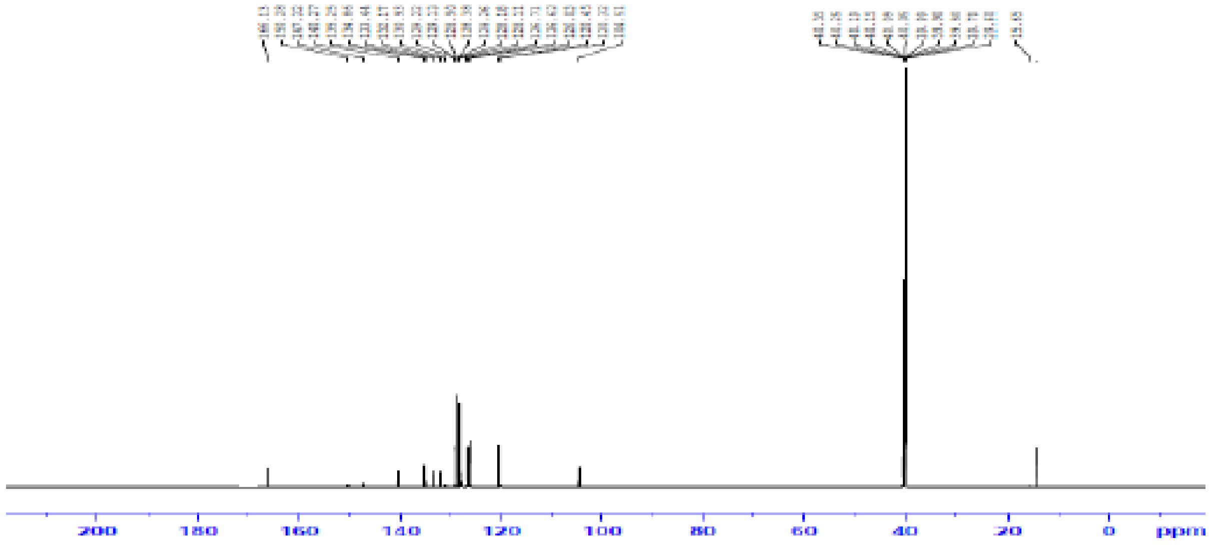

IR of compound **5a**

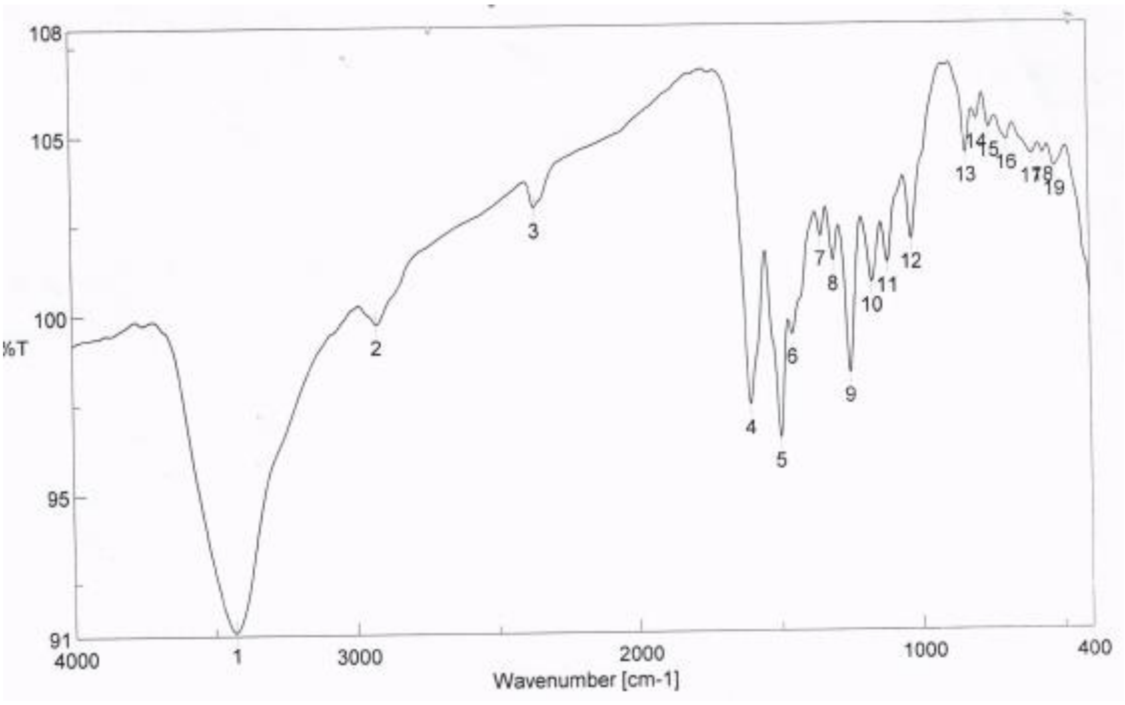

[Result of Peak Picking]

No.	Position	Intensity	No.	Position	Intensity	No.	Position	Intensity
1	3429.78	91.1012	2	2924.52	99.7181	3	2360.44	102.935
4	1601.59	97.3999	5	1497.45	96.4665	6	1453.1	99.3655
7	1351.86	102.08	8	1307.5	101.383	9	1250.61	98.2773
10	1171.54	100.786	11	1114.65	101.332	12	1029.8	101.983
13	832.133	104.381	14	792.6	105.307	15	749.209	105.072
16	687.498	104.722	17	598.789	104.33	18	559.255	104.35
19	518.758	103.967						

MS of compound **5a**

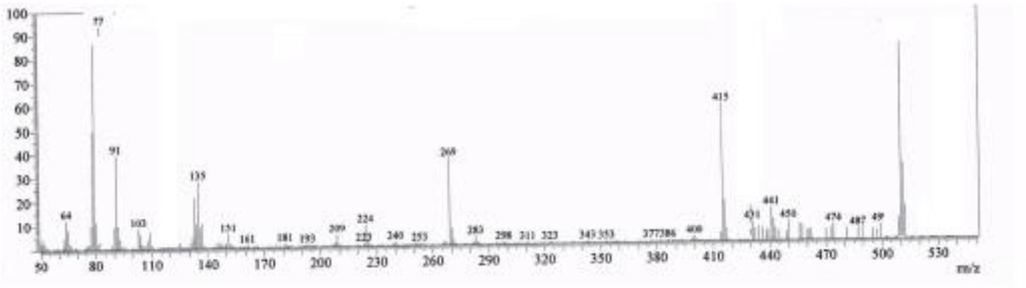

Characterization of chitosan-SrO nanocomposite

Figure S2. Comparative FTIR spectra of chitosan (A) and chitosan-strontium oxide nanocomposite (B) (10% wt.)

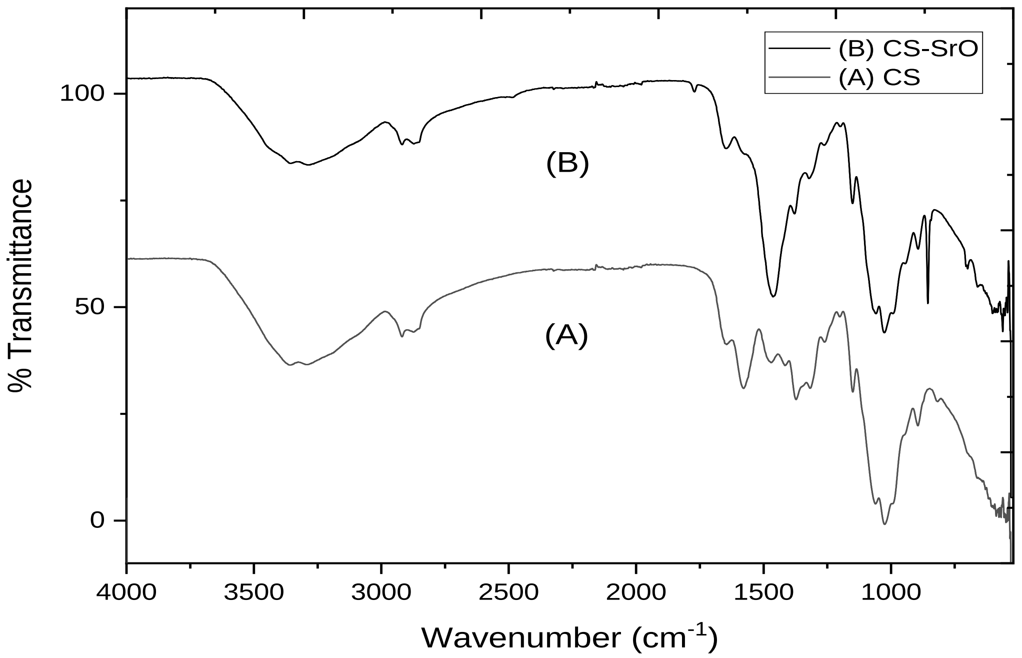

Figure S3. FESEM of chitosan (A), strontium oxide nanoparticles (B), and chitosan-SrO composite, 10% wt. (C).

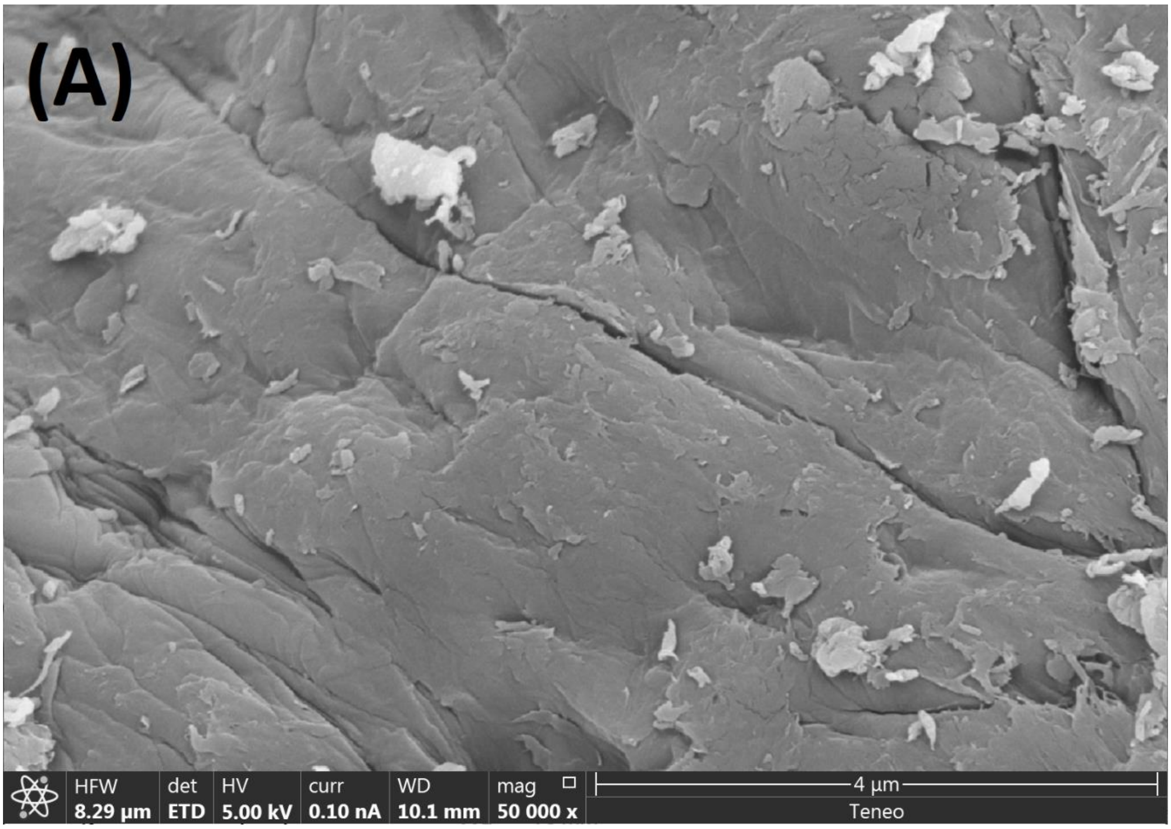

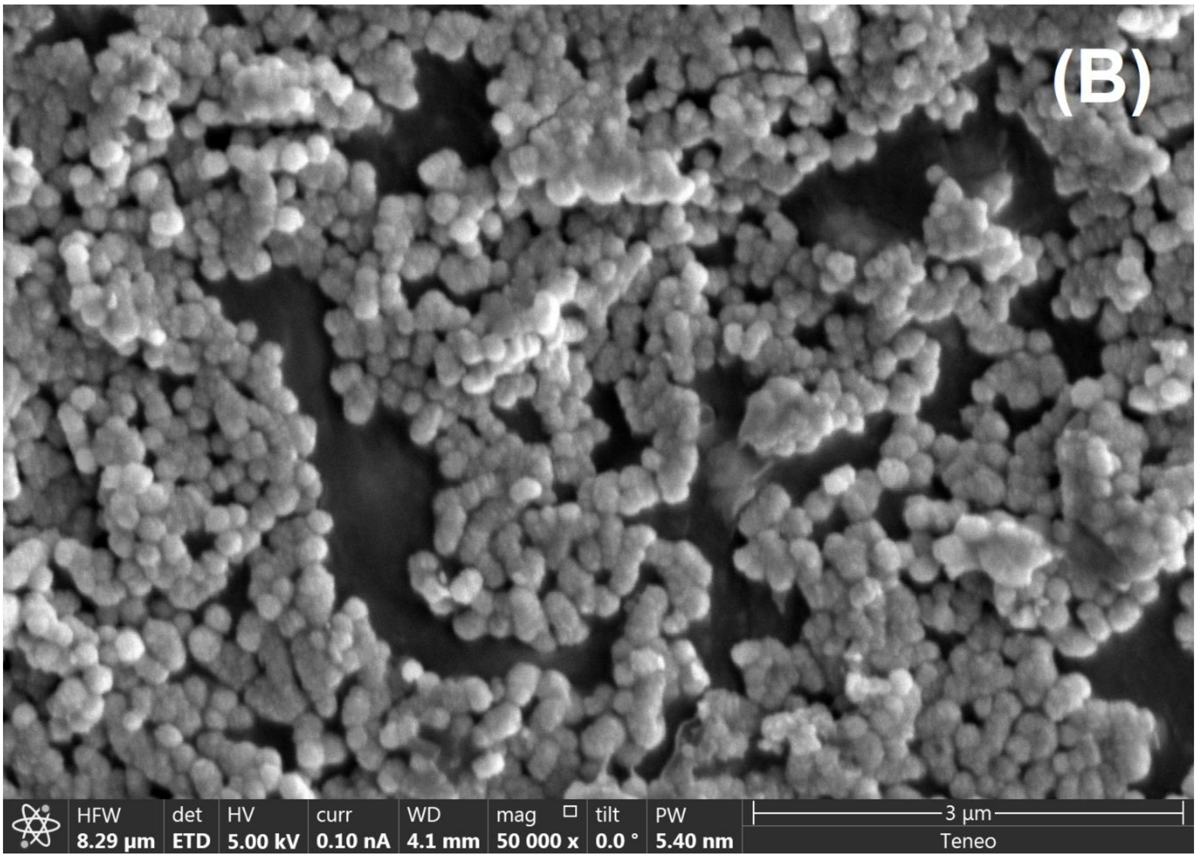

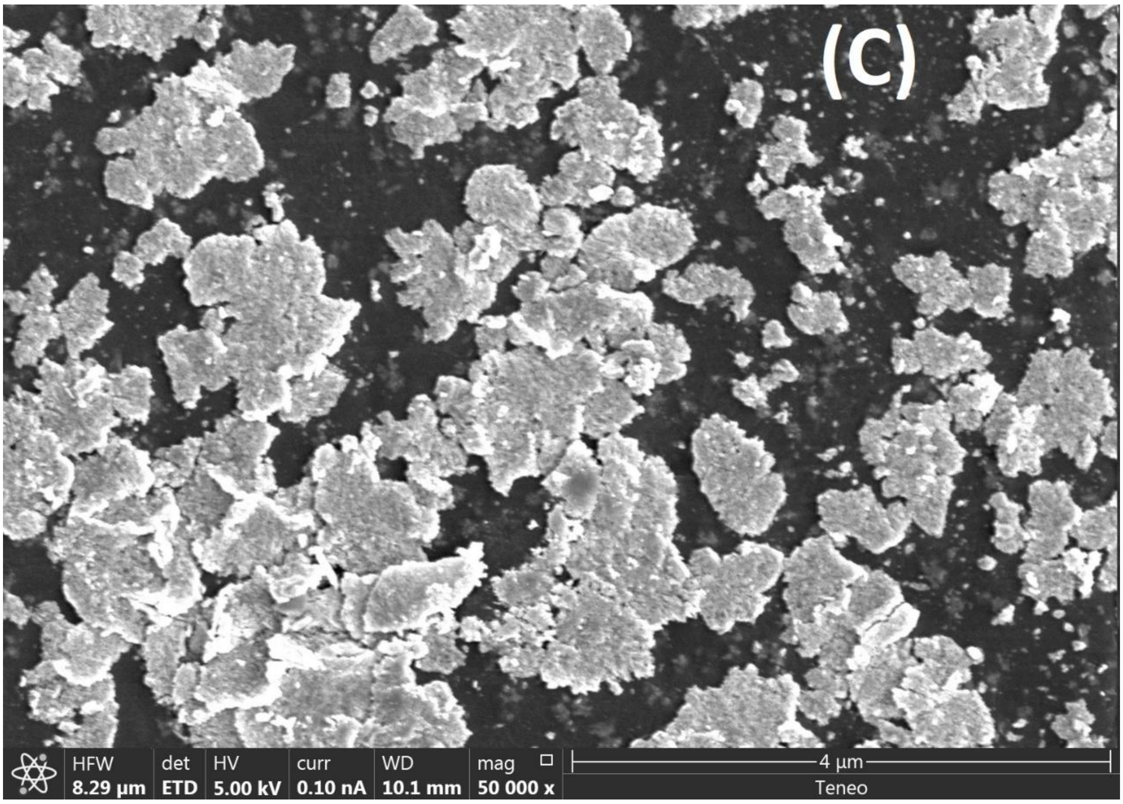

Figure S4. Energy Despersive Spectroscopy of chitosan-SrO nanocomposites

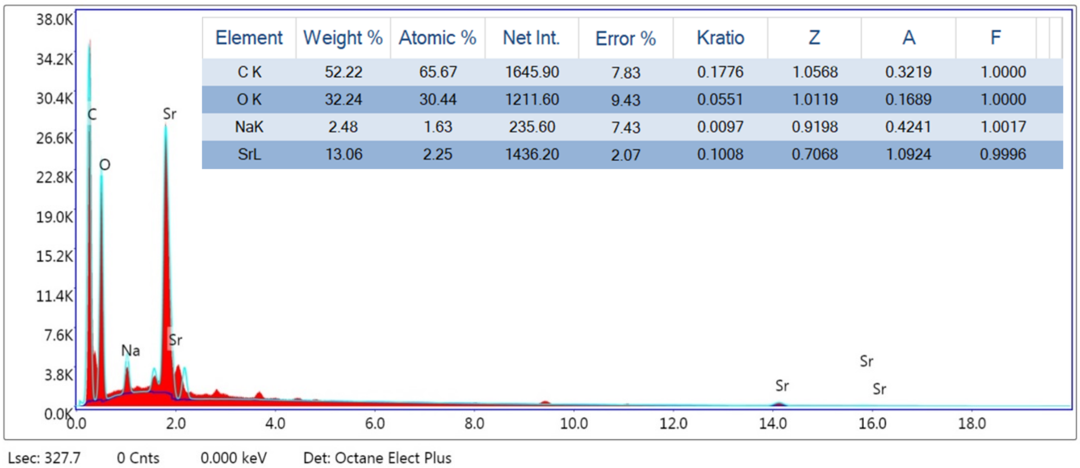

Element	Weight %	Atomic %	Net Int.	Error %	Kratio	Z	A	F
C K	52.22	65.67	1645.90	7.83	0.1776	1.0568	0.3219	1.0000
O K	32.24	30.44	1211.60	9.43	0.0551	1.0119	0.1689	1.0000
NaK	2.48	1.63	235.60	7.43	0.0097	0.9198	0.4241	1.0017
SrL	13.06	2.25	1436.20	2.07	0.1008	0.7068	1.0924	0.9996
